# Effect of prenatal administration of venlafaxine on postnatal development of rat offspring

**DOI:** 10.2478/v10102-012-0016-3

**Published:** 2012-06

**Authors:** Michal Dubovický, Eszter Császárová, Zuzana Brnoliaková, Eduard Ujházy, Jana Navarová, Mojmír Mach

**Affiliations:** Institute of Experimental Pharmacology & Toxicology, Slovak Academy of Sciences, SK-84104 Bratislava, Slovakia

**Keywords:** selective serotonin re-uptake inhibitors and/or serotonin, noradrenaline re-uptake inhibitors, venlafaxine, pregnancy, fetus, pups, postnatal development, rat

## Abstract

About 3% of pregnant women are treated with antidepressant drugs during gestation. After delivery the number of treated women increases to 5 to 7%. Most prescribed antidepressants in pregnancy are selective serotonin re-uptake inhibitors and/or serotonin and noradrenaline re-uptake inhibitors, such as fluoxetine, paroxetine, sertraline, citalopram and venlafaxine (VENF). Despite the fact that VENF has been assigned to pregnancy category C by the FDA, experimental studies with this drug are rare. The aim of this pilot study was to investigate the effect of prenatal administration of VENF on early postnatal development of rat offspring and selected biochemical variables at weaning of pups. Pregnant female Wistar rats were treated with VENF from day 15 to 20 of gestation at the doses of 7.5, 37.5 and 70 mg/kg. Females were allowed to spontaneously deliver their pups. After delivery the pups were inspected for viability, gross malformation and they were weighed on day 0, 4 and 21 *post partum*. On day 21 *post partum*, the pups were killed, brains were removed from the skulls and blood samples were collected for biochemical assay (proteins, glucose-GOD, glucose-HEX, lactate dehydrogenase, aspartate aminotransferase, alanine aminotransferase and total antioxidant status). The study showed that prenatal VENF administration resulted in a mild maternal intoxication manifested by decreased body weight gain of pregnant females. There was no effect of the drug tested on the body and brain weights of offspring. No obvious morphological alterations were observed in the delivered pups. Similarly, there were no changes in the selected biochemical variables determined.

## Introduction

Affective disorder represents a serious health issue of modern society. Many women, reproductive age not excluded, suffer from major depression or bipolar disorder and in certain cases pregnancy and/or delivery may release serious affective disorders. The estimated prevalence of depression and other mood disorders in pregnancy ranges from 9 to 16% (Evans *et al.,* 2001; Melville *et al*, 2010).

The major dilemma for gynecologists and obstetricians is to treat or not to treat depression in pregnancy. Consequences of untreated depression during pregnancy can be so serious that the benefit of antidepressant therapy may overweigh the possible risk for injury of fetal and/or neonatal development. Untreated depression represents a risk mostly for pregnant women. There is a higher risk of maternal morbidity, including arterial hypertension leading to preeclampsia or eclampsia, suicide attempts and *post partum* depression. Depression may be associated also with an increased risk of preterm or operative delivery, low birth weight, irritability, prematurity, sleep disorders, admission of the newborn to the neonatal intensive care unit and/or increased level of internalizing behaviors in childhood (Chung *et al*., 2004; Oberlander *et al*., 2010).

About 2 to 3% of pregnant women are treated with antidepressant drugs during gestation. After delivery the number of treated women increases to 5 to 7%. Most prescribed antidepressants in pregnancy and lactation are selective serotonin re-uptake inhibitors and/or serotonin and noradrenaline re-uptake inhibitors (SSRIs/SNRIs), such as fluoxetine, paroxetine, sertraline, citalopram and venlafaxine (Nonacs & Cohen, 2002; Tuccori *et al*., 2009; Freeman, 2011). SSRIs act mostly by inhibiting the re-uptake of serotonin after being released into synapses. In addition, several other mechanisms are suggested for the desired effect, *e.g.* neuroprotection and anti-inflammatory and immunomodulatory effects. SSRIs act on signal pathways such as cyclic AMP on the postsynaptic neuronal cell, which leads to the release of the brain derived neurotrophic factor (BDNF). BDNF, in turn, enhances the growth and survival of cortical neurons and synapses (Pilar-Cuéllar *et al.,* 2012). SNRIs work by inhibiting the reuptake of both serotonin and noradrenaline. This results in an increase in the extracellular concentrations of serotonin and noradrenaline and therefore an increase in their neurotransmission (Papakostas *et al.,* 2007). As the SSRI/SNRI drugs substantially cross the placenta (Rampono *et al*., 2009) and get into breast milk (Bellantuono *et al*., 2007), they can enter the developing brain and interfere with monoaminergic neurotransmission, which plays a key role in brain development. Therefore, this kind of drugs can represent a risk for the developing brain and postnatal neurobehavioral development. These drugs do not represent a risk factor for the developing embryo/fetus from the point of view of serious structural malformations. However, they can cause a transient decrease in uterine blood flow followed by hypoxia/ischemia and changes in fetal breathing, brain electrical activity and/or hypothalamus-pituitary-adrenal axis function. The SSRI drugs used in the 3^rd^ trimester were found to increase the risk for neonatal behavioral syndrome, pulmonary hypertension, abortions and delayed neuromotor and behavioral development of children (Moses-Kolko *et al.*, 2005Moses-Kolko *et al.*, 2010; Gentile, 2005). Rare experimental studies showed the effect of fluoxetine on functional development of the frontal lobe of the cortex and the middle brain as well as on neurobehavioral development of offspring (Morrison *et al*., 2005). The SSRI/SNRI drugs can affect functional development of serotonergic and noradrenergic neurotransmitter systems. In turn, they can affect the development of the neuronal circuit with consequent manifestation of maladaptive behavior, which can persist until maturation, adulthood and/or even senescence as depression, anxiety, autism, and/or aggressive behavior.

Venlafaxine (VENF) belongs to the group of SNRI antidepressant drugs. It affects both serotonin and noradrenaline or even dopamine neurotransmission (Weikop *et al*., 2007). The mechanism of the antidepressant action of VENF in humans is believed to be associated with its potentiation of neurotransmitter activity in the CNS. Preclinical studies have shown that VENF and its active metabolite O-desmethylvenlafaxine are potent inhibitors of neuronal serotonin and norepinephrine reuptake and weak inhibitors of dopamine reuptake (Wellington and Perry, 2001). VENF has been assigned to pregnancy category C by the FDA. The FDA has classified VENF as category C with regard to pregnancy risk. This means that there have not been well-controlled studies in humans examining safety or that animal studies have demonstrated adverse effects to the developing fetus (Patil *et al*., 2011). Increased monoamine levels in the developing brain due to prenatal and perinatal VENF may interfere with functional maturation of the brain and increase the risk for neurobehavioral and mental disorders.

The aim of this pilot study was to investigate the effect of prenatal administration of VENF on early postnatal development of rat offspring and selected biochemical variables at weaning of pups.

## Material and Methods

### Animals

Female Wistar rats (initial weight 150–190 g, age 3–4 months, n=60) obtained from the breeding station of the Institute of Experimental Pharmacology and Toxicology, Slovak Academy of Sciences, Dobra Voda, Slovak Republic (reg. No. SK CH 24 011), were used in the study. After 14 days of acclimatization, the females were mated with males in the ratio 1 male : 3 females. The presence of spermatozoa in vaginal smears was considered day 0 of gestation. Pregnant rats, 3 per cage, were housed in plastic cages with wooden shavings for bedding. On day 15 of gestation the females were separated and housed individually. The animals had free access to food pellets and water and were kept in a temperature and humidity controlled room (20–24 °C, relative humidity 50–70%) with 12/12 hrs light/dark cycle. The experiments were conducted in compliance with the Principles of Laboratory Animal Care issued by the Ethical Committee of the Institute of Experimental Pharmacology and Toxicology, Slovak Academy of Sciences, Bratislava, and the experimental design was approved by the State Veterinary and Food Administration of the Slovak Republic.

### Venlafaxine treatment

Venlafaxine hydrochloride (VENF) is a antidepressant for oral administration. It is designed as (R/S)-1-[2-(dimethylamino)-1-(4-methoxyphenyl)ethyl] cyclohexanol hydrochloride or (±)-1-[a-[(dimethyl-amino)methyl]-p-methoxybenzyl] cyclohexanol hydrochloride and has the empirical formula of C_17_H_27_NO_2_ HCl. Its molecular weight is 313.87 (Chemoz, Czech Republic, purity, 98.5%). VENF dissolved in distilled water was orally administered to pregnant rats from day 15 to 20 of gestation at doses of 7.5, 37.5 and 75 mg/kg in the application volume 0.5 ml/100 g of body weight (n = 4-9 pregnant females/group). The animals from the control group received distilled water as vehiculum.

### Postnatal inspection of offspring

Pregnant females were allowed to spontaneously deliver their pups. The delivered pups were inspected for viability and possible gross malformations, number and individual weights of the pups were recorded to protocol. On day 4 *post partum* (PP), each litter was subjected to “culling” procedure, *i.e.* the number of pups in each litter was reduced to 8, 4 males and 4 females per litter, and the individual weights of the culled pups were recorded. On day 21 PP, the animals were weighed and sacrificed by decapitation, blood samples were collected for biochemical assay, the brains were removed from the skulls, weighed and frozen for further investigation.

### Biochemical study

A part of the experimental animals of both sexes from the highest dose group and the control group were used in this study. The overal physiological status of the experimental animals was assessed by the following biochemical variables: blood levels of glucose (GLU), lactate dehydrogenase (LDH), aspartate aminotransferase (AST) and alanine aminotransferase (ALT) were measured by commercially available diagnostic kits (DiaSys Diagnostic Systems, Germany). Total antioxidant status (TAS) was also assessed (Randox Life Sciences, Great Britain). All the blood samples were processed on the BioLis 24i chemistry analyzer (Tokyo Boeki Machinery Ltd., Tokyo, Japan) according to the manufacturer′s instructions.

### Statistics

Raw data were analyzed by means of relevant statistical analysis (one-way ANOVA, factorial ANOVA, repeated measures ANOVA and non-parametric t-test). Differences were considered statistically significant when *p≤*0.05. The values are expressed as means ± S.E.M or S.D.

## Results

### Maternal toxicity

**Figure 1:**
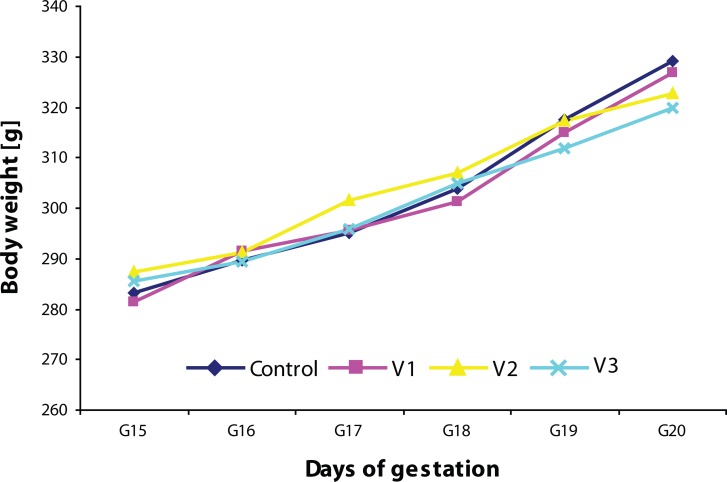
Effect of prenatal VENF on body weight of pregnant females. G – gestation, V1 – VENF at dose 7.5 mg/kg, V2 – VENF at dose 37.5 mg/kg, V3 – VENF at dose 70 mg/kg; *p<*0.01 – statistically significant difference, repeated measures ANOVA (effect of time).

**Figure 2:**
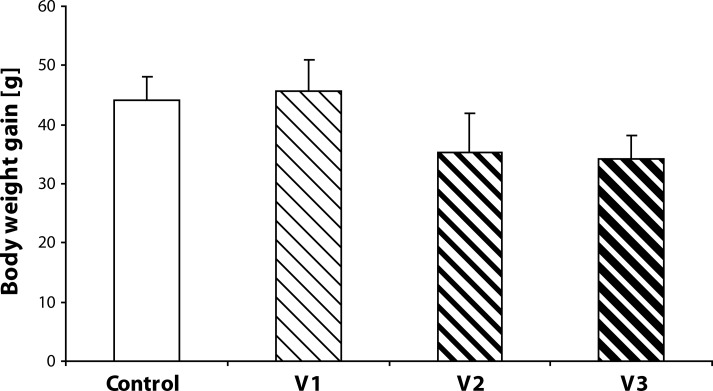
Effect of prenatal VENF on body weight gains of pregnant females. G – gestation, V1 – VENF at dose 7.5 mg/kg, V2 – VENF at dose 37.5 mg/kg, V3 – VENF at dose 70 mg/kg.

During the experiment, no pregnant female aborted or died. There was no effect of the treatment on the duration of pregnancy. General health status of the pregnant rats was good. The repeated measures ANOVA demonstrated asignificant effect of time on the body weight of pregnant rats with a significant increase of weight from days 15 to 20 of gestation, [F(5,160)=150.88; *p<*0.0001]. There was no significant effect of VENF treatment on the body weight of females [F(3,32)=0.04191; *p=*0.988]. Mean body weight of pregnant females treated with the highest dose of VENF on days 19 and 20 were somewhat lower compared to controls, however the differences failed to be statistically significant (Figure 1). Figure 2 shows the body weight gains from day 15 to 20 of gestation. Mean values in this variable were lower in the pregnant females treated with the middle and the highest doses of VENF compared to controls (approximately 20%), however, the differences failed to be statistically significant.

### Offspring

**Figure 3:**
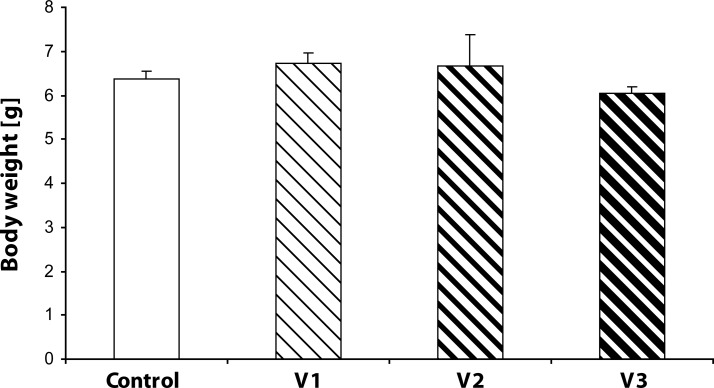
Effect of prenatal VENF on body weight of pups on day 0 *post partum*. V1 – VENF at dose 7.5 mg/kg, V2 – VENF at dose 37.5 mg/kg, V3 – VENF at dose 70 mg/kg.

**Figure 4:**
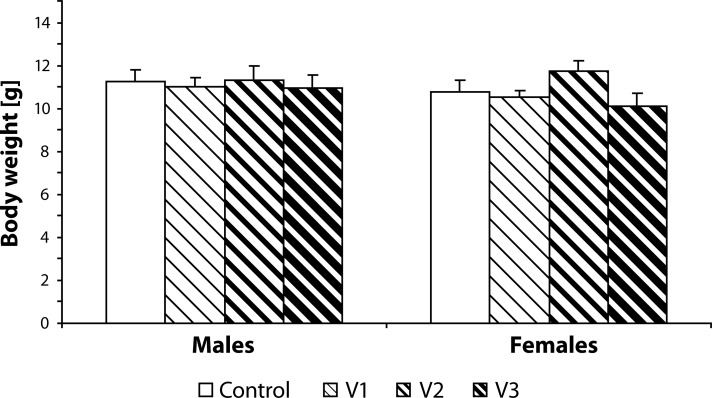
Effect of prenatal VENF on body weight of pups on day 4 *post partum*. V1 – VENF at dose 7.5 mg/kg, V2 – VENF at dose 37.5 mg/kg, V3 – VENF at dose 70 mg/kg.

**Figure 5:**
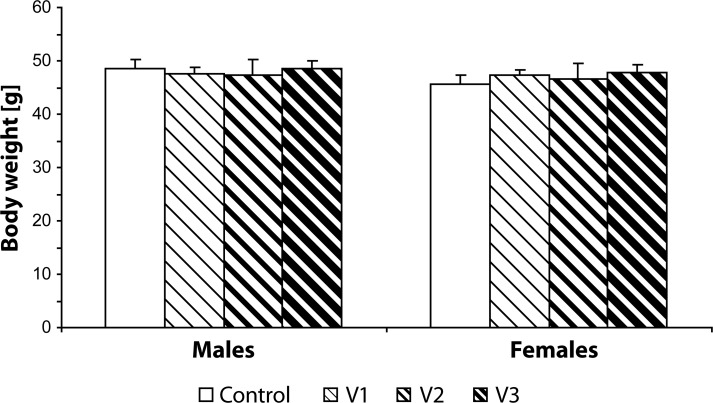
Effect of prenatal VENF on body weight of pups on day 21 *post partum*. V1 – VENF at dose 7.5 mg/kg, V2 – VENF at dose 37.5 mg/kg, V3 – VENF at dose 70 mg/kg.

**Figure 6:**
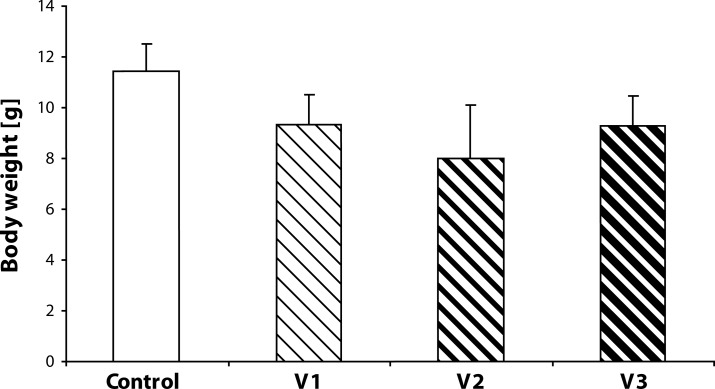
Effect of prenatal VENF on the number of pups on day 0 *post partum*. V1 – VENF at dose 7.5 mg/kg, V2 – VENF at dose 37.5 mg/kg, V3 – VENF at dose 70 mg/kg.

**Figure 7:**
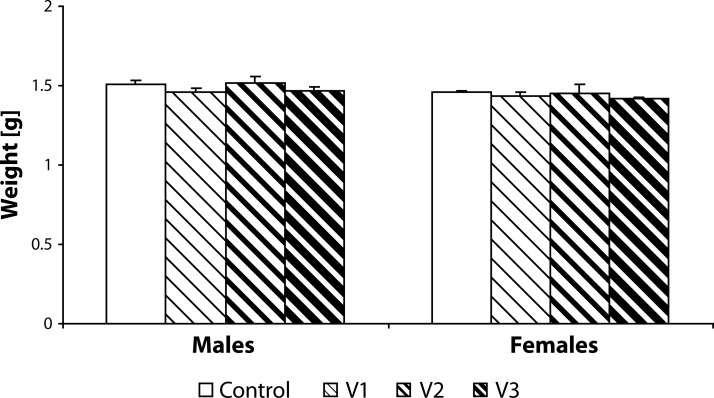
Effect of prenatal VENF on brain weight of pups on day 21 *post partum*. V1 – VENF at dose 7.5 mg/kg, V2 – VENF at dose 37.5 mg/kg, V3 – VENF at dose 70 mg/kg.

Statistical analysis did not demonstrate any statistically significant effect of VENF treatment on the body weight of pups measured on days 0, 4 and 21 PP (Figures 3–5). Not even, on days 4 and 21 PP when male and female pups were weighed separately, there were no significant differences between males and females. In the groups with the middle and the highest doses, there was one cachectic pup/litter which later died. Concerning the number of pups/litter, in the litters from females treated with VENF, there was a decrease in the mean number of pups compared to controls. However, the differences were not statistically significant (Figure 6). In two mothers treated with the highest dose of VENF, cannibalism was observed. Two-way ANOVA revealed no significant effect of treatment on brain weight, yet there was a significant effect of gender with significantly higher mean values of brain weight in males compared to females; males: 1.48 ± 0.01, females: 1.45 ± 0.01 [F(1, 50)=4.175; *p=*0.046] (Figure 7).

### Biochemical variables

None of the biochemical variables assessed showed any significant alteration in the group with the highest VENF dose compared to controls (Table 1).

**Table 1:** Effect of prenatal VENF administration on selected biochemical variables.

	Control	VENF
PROT	45.47 ± 5.01	48.13 ± 3.73
LDH	28.22 ± 6.49	28.70 ± 10.44
ALAT	1.14 ± 0.21	1.11 ± 0.19
ASAT	5.29 ± 1.04	5.47 ± 1.97
GLU-GOD	9.11 ± 0.76	8.95 ± 0.60
GLU-HEX	8.78 ± 0.67	8.94 ± 0.52
TAS	3.02 ± 0.26	2.99 ± 0.87

PROT – proteins, GLU – glucose, LDH – lactate dehydrogenase, AST – aspartate aminotransferase, ALT – alanine aminotransferase, TAS – total antioxidant status.

## Discussion

Our study showed that prenatal VENF administration resulted in a mild maternal intoxication manifested by decreased body weight gain of pregnant females. There was no effect of the drug tested on body and brain weights of offspring. No obvious morphological alterations were observed in the delivered pups. Similarly, there were no changes in the selected biochemical variables determined.

A variety of behavioral and neurological symptoms including irritabilty, persistent crying, shivering, tremor, restlessness, feeding difficulties, sleep disorders, and seizures, have been reported in infants born to women who used SSRIs during pregnancy (Moses-Kolko *et al*., 2005). This clinical picture has been interpreted as a neonatal withdrawal syndrome (or direct toxic effect of antidepressants on the newborn) and desiganted neonatal behavioral syndrome. This syndrome includes poor tone, resipratory distress and hypoglycemia (Favrelière *et al*., 2010). Of the unwanted effects of SSRIs in pregnancy, Gentile (2005) reported subtle effects on motor development and motor control.

As neonatal behavioral syndrome after SSRIs withdrawal was found to be associated with hypoglycemia, we decided to investigate the basic biochemical profile of the rat offspring exposed prenatally to VENF. We did not find any marked effect of VENF on these variables. Abnormal GLU level might indicate metabolic disturbances, however in our experiments it oscillated within the physiological range. Neither was LDH, often used as a marker of tissue breakdown, changed. AST/ALT ratio is generally used in differentiating between causes of liver damage insinuating possible hepatotoxicity (Sumner *et al*., 2010). No such impairment was observed. Finally, the results showed that the mean serum TAS did not differ significantly compared to controls.

Prenatal administration of VENF caused non-significant decrease in the body weight gain of pregnant females treated with the middle and highest doses of VENF. Nevertheless, from the toxicological point of view we consider these decreases to be relevant. The OECD Guideline No. 426 on Developmental Neurotoxicity Study recommend to choose the highest dose level with the aim to induce some signs of maternal toxicity, such as decreased body weight gain, yet not more than 10% (OECD, 1995). However, the relatively lower number of delivered pups in VENF exposed animals might be a reason of decreased body weight gains. It can not be excluded either that the decreased body weight gain in the mothers treated with the highest dose of VENF was also associated with a higher level of distress, manifested by individual cases of cannibalism (DeSantis and Schmaltz, 1984).

Our results related to the weight of pups are in contrats to the developmenatal study with both fluoxetine and venlafaxine (da-Silva *et al.,* 1999). They reported reduced body weight of litters, however by the time of weaning, the weight of litters from treated dams was equal to the weight of control litters. The different senzitivity of the rat strain used in this study may offer a tentative explanation of the weight divergencies.

Although, VENF has been assigned to pregnancy category C by the FDA, experimental studies with VENF are rare. Early exposure (pre-, peri- and/or neonatal periods) to VENF or other SSRIs/SNRIs may disrupt the normal maturation of the monoaminergic system and neuronal processes dependent on it. Functional alterations of brain development, in turn, can lead to various neurobehavioral dysfunctions manifested in later postnatal life (Dubovický *et al*., 2010). The SSRIs/SNRIs are the most common antidepressant drugs used in treatment of mood disorders in pregnancy and the postpartum period. The serotonergic system plays an important role in brain development. Thus, abnormal stimulation of serotonin receptors, as a result of increased synaptic availability of serotonin, and disruption of serotonin transporters activity during brain development due to SSRIs/SNRIs can lead to functional alterations followed by postnatal neurobehavioral dysfunctions. Delayed and/or late neurobehavioral consequences of action of prenatal and perinatal insults, including antidepressants, occur mostly in late postnatal development, in adolescence, adulthood or even senescence. Some of these dysfunctions can be hidden or masked. They can be stress-related, manifesting in response to stressful stimuli (Dubovický *et al*., 2008).

Effects of preventing re-uptake of presynaptic serotonin on fetal and postnatal development have not been sufficiently elucidated. Moreover, prospective epidemiological studies focused on older children, adolescents and adults exposed *in utero* to antidepressants are not available. Understanding of the role of monoamines in brain development is also important to identify the possible adverse effects of SSRIs/SNRIs exposure during pregnancy and lactation. Insight into the potential functional and behavioral consequences of the alterations induced by these drugs remains to be provided. Thus, scientists should focus on late consequences of prenatal and perinatal exposures with emphasis on various patterns of behavior, including emotions, aggression, sociability, coping and sexual functions.

In conclusion, the present study showed that VENF at the doses 37.5 and 70 mg/kg provoked acertain kind of maternal toxicity manifested by decreased body weight. Prenatal administration of VENF did not affect early postntal development of the rat offspring. However, possible effects of the drug tested on functional brain development in relation to postnatal neurobehavioral development should be followed up. This will be the aim of our further research.
